# Impact of Graft-Resident Leucocytes on Treg Mediated Skin Graft Survival

**DOI:** 10.3389/fimmu.2021.801595

**Published:** 2021-11-29

**Authors:** Romy Steiner, Anna M. Weijler, Thomas Wekerle, Jonathan Sprent, Nina Pilat

**Affiliations:** ^1^ Department of General Surgery, Medical University of Vienna, Vienna, Austria; ^2^ Immunology Division, Garvan Institute of Medical Research, Sydney, NSW, Australia; ^3^ St Vincent’s Clinical School, University of New South Wales, Sydney, NSW, Australia

**Keywords:** transplantation, allo-recognition, Regulatory T cells (Tregs), tolerance, IL-2 complexes, passenger leucocytes, graft-resident leucocytes

## Abstract

The importance and exact role of graft-resident leucocytes (also referred to as passenger leucocytes) in transplantation is controversial as these cells have been reported to either initiate or retard graft rejection. T cell activation to allografts is mediated *via* recognition of intact or processed donor MHC molecules on antigen-presenting cells (APC) as well as through interaction with donor-derived extracellular vesicles. Reduction of graft-resident leucocytes before transplantation is a well-known approach for prolonging organ survival without interfering with the recipient’s immune system. As previously shown by our group, injecting mice with IL-2/anti-IL-2 complexes (IL-2cplx) to augment expansion of CD4 T regulatory cells (Tregs) induces tolerance towards islet allografts, and also to skin allografts when IL-2cplx treatment is supplemented with rapamycin and a short-term treatment of anti-IL-6. In this study, we investigated the mechanisms by which graft-resident leucocytes impact graft survival by studying the combined effects of IL-2cplx-mediated Treg expansion and passenger leucocyte depletion. For the latter, effective depletion of APC and T cells within the graft was induced by prior total body irradiation (TBI) of the graft donor. Surprisingly, substantial depletion of donor-derived leucocytes by TBI did not prolong graft survival in naïve mice, although it did result in augmented recipient leucocyte graft infiltration, presumably through irradiation-induced nonspecific inflammation. Notably, treatment with the IL-2cplx protocol prevented early inflammation of irradiated grafts, which correlated with an influx of Tregs into the grafts. This finding suggested there might be a synergistic effect of Treg expansion and graft-resident leucocyte depletion. In support of this idea, significant prolongation of skin graft survival was achieved if we combined graft-resident leucocyte depletion with the IL-2cplx protocol; this finding correlated along with a progressive shift in the composition of T cells subsets in the grafts towards a more tolerogenic environment. Donor-specific humoral responses remained unchanged, indicating minor importance of graft-resident leucocytes in anti-donor antibody development. These results demonstrate the importance of donor-derived leucocytes as well as Tregs in allograft survival, which might give rise to new clinical approaches.

## Introduction

T cell reactions to organ allografts are directed largely to major histocompatibility (MHC) antigens expressed on donor cells, both within the graft and on graft-resident leucocytes; these “passenger leucocytes” migrate from the graft to the periphery to activate host T cells (1). In part, these reactions reflect “direct” contact of T cells with intact donor MHC antigens on graft-derived antigen-presenting cells (APCs), mostly dendritic cells (DC) (2). In parallel, T cells also make “indirect” contact with donor antigens as the result of degradation of their MHC molecules into specific donor peptides that bind to host MHC molecules displayed on host APC. Although both direct and indirect pathways of allorecognition are involved in graft rejection, the relative importance of these two pathways is still controversial (3).

The theory of “passenger leucocytes” as a target for allorecognition was first suggested in 1957 by G. D. Snell who described a highly immunogeneic cell population within the parenchyma and stroma of a solid organ allograft in mice (4). Thereafter, it was shown that graft immunogenicity can be attenuated by reduction of passenger leucocytes, *via* either prior organ culture *in vitro* or pre-transplantation on host-type mice. With both approaches, depletion of passenger leucocytes retarded rejection and thus prolonged survival of skin allografts in rodent models (5, 6). Likewise, studies of heart allograft transplantation in rats showed prolongation of survival if donor and/or organ were pretreated with a combination of photochemicals, total body irradiation (TBI) and injection of antilymphocyte globulin (7, 8).

Notably, Barker and Billingham demonstrated in 1968 that allograft survival was prolonged if donor cell trafficking through lymphatic vessels was inhibited (9). This finding suggested that sensitization of recipient T cells depended crucially on graft-resident leucocytes leaving the organ shortly after transplantation. In this respect, recovery of lymphatic vessels severed during skin transplantation surgery takes 5 to 7 days, thus impeding migration of donor cells to the draining lymph nodes (10). More recently, Marino et al. (11) suggested a different mechanism independent of intact donor APC, namely responses to donor-derived extracellular vesicles (EVs). Here, it was demonstrated that trafficking of EVs from the graft into recipient lymph nodes resulted in host cells expressing donor intact (rather than processed) MHC molecules alongside self MHC as early as 12 hours after transplantation. These allo-MHC “cross-dressed” recipient APCs efficiently activated alloreactive T cells in a skin transplantation setting. In addition, it was recently shown that donor-derived dendritic cells (DCs) are a major source of exosomes capable of promoting allograft rejection in a murine heart transplant model (12). This mechanism was originally suggested by Herrera et al. in 2004 where cross-dressing of DCs with donor MHC was described as a third pathway of allo-recognition, termed the “semi-direct” pathway (13). This pathway of acquired absorption of intact MHC-bearing membrane vesicles is now termed trogocytosis (14) and dates back to studies on cell-membrane exchange in the 1970s (15).

Despite the well-established migration of graft-derived APC and EVs to the draining lymph nodes, it is important to emphasize that shedding of EVs is not an exclusive property of APC and so may also arise from parenchymal cells in the graft. For this reason, the precise role of donor-derived leucocytes in transplantation continues to be controversial (16, 17). Here we have investigated the graft-resident leucocytes in a skin allograft transplantation setting largely devoid of passenger leucocytes, including T cells. We utilized our prior finding that injecting mice with IL-2/antibody complexes (IL-2cplx) led to Treg expansion and prolonged survival of skin allografts. Notably, a proportion of the expanded Tregs entered the grafts, raising the question whether these immigrant Tregs contributed to graft survival. To investigate this question, we examined the effects of irradiating skin allografts to deplete APC and T cells before transplantation (18), followed by IL-2cplx treatment of the host to promote host Treg entry into the grafts. In brief, the results show that, in combination, these procedures significantly enhance graft survival.

## Results

### Depletion of Skin-Resident Leucocytes After TBI

**Figure 1:**
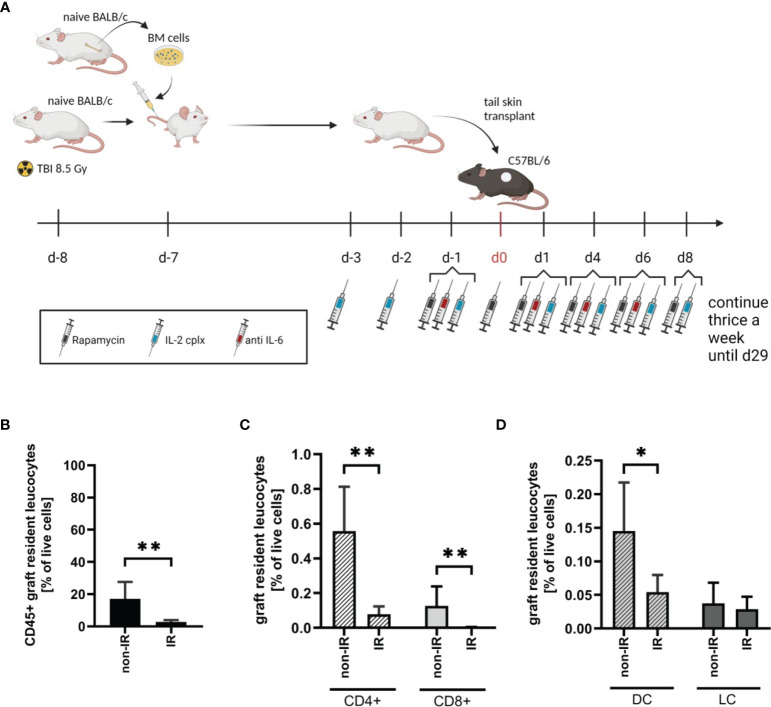
Effective reduction of donor tail skin resident leucocytes 8 days after exposure to lethal total body irradiation. **(A)** Design of an allograft transplantation setting with reduced passenger leucocytes and IL-2 complex protocol treatment schema for indicated groups of skin allograft recipients. Donor mice were lethally irradiated (8.5Gy) d-8 and reconstituted with bone marrow cells of a naïve BALB/c mouse d-7. Donor tail skin was transplanted d0 onto recipient C57BL/6 mice. Indicated recipient mice in addition received a combination of IL-2 complexes, Rapamycin and a short term treatment of anti-IL-6. **(B)** Frequency of graft-resident CD45+ leucocytes within irradiated (IR; n = 7) and non-irradiated (non-IR; n = 4) skin grafts at time point of transplantation (d0) (mean 3% IR *vs* 17% non-IR; p = 0.006). **(C)** Graft-resident T-cell subsets within irradiated (n = 7) and non-irradiated (n = 4) skin grafts d0. Frequency of CD4+ (mean 0.079% IR *vs* 0.56% non-IR; p = 0.006) and CD8+ (mean 0.004% IR *vs* 0.13% non-IR; p = 0.006) T-cells within total graft-resident cells. **(D)** Frequency of graft-resident dendritic (CD45+ MHCII+ CD11c+; DC; mean 0.05% IR *vs* 0.15% non-IR; p = 0.03) and Langerhans cells (CD45+ MHCII+ CD11b+; LC; mean 0.03% IR *vs* 0.04% non-IR; NS) within IR and non-IR skin grafts d0 (n = 5 each). Analysis **(B–D)** was performed using flow cytometry and mean percentages are shown. Error bars indicate SD. (*P < 0.05; **P < 0.01; two-tailed t test with unequal variances).

To investigate the effect of passenger leucocytes on skin allograft survival, we established a fully-mismatched, clinically-relevant murine transplantation model (BALB/c on C57BL/6) largely depleted of graft-resident bone-marrow (BM)-derived cells. Donor mice (BALB/c) were subjected to lethal TBI (8.5 Gy) at day 8 prior to skin graft donation and were reconstituted with bone marrow (BM) cells from isogenic donors at day -7 (**Figure 1A**) (18). When irradiated (IR) donor skin is compared to non-irradiated (non-IR) skin at the time point of skin donation, percentages of leucocytes (identified as CD45+ cells amongst total viable skin cells) were significantly reduced (**Figure 1B**). There was comparable 80-90% depletion of CD4+ and CD8+ T cell frequencies (**Figure 1C**) and ~70% depletion of (dermal) DCs (CD45+ MHCII+ CD11c+), with a parallel reduction of LCs (CD45+ MHCII+ CD11b+) though this difference was not significant (**Figure 1D**). These data confirm the effective depletion of skin-resident leucocytes in the tail skin of donor mice subjected to IR (day 8 post IR).

### IR Exposure Leads to Elevated Early Graft Infiltration of Recipient Leucocytes in Naïve but Not IL-2cplx Treated Host Mice

**Figure 2:**
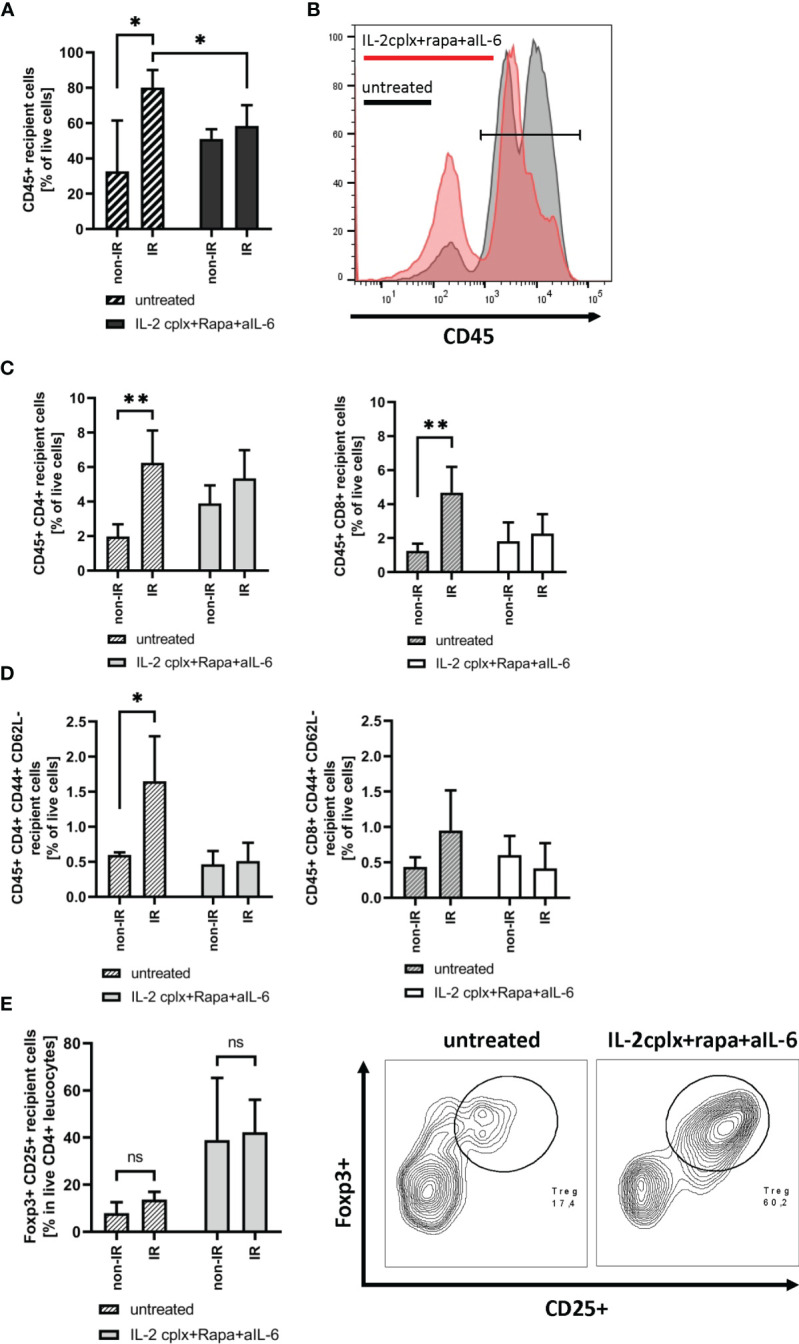
Analysis of graft infiltrating leucocytes (recipient) 6 days post transplantation. **(A)** Frequency of CD45+ graft infiltrating leucocytes within skin grafts that were irradiated (IR) *vs*. non-irradiated (non-IR) before transplantation on mice receiving IL-2 complexes in combination with rapamycin and a short course of anti-IL-6 (IR: n = 5; non-IR: n = 6) or left untreated (IR: n = 5; non-IR: n = 5). **(B)** Representative histogram showing frequency of CD45+ graft infiltrating leucocytes in previously irradiated skin allografts. Results for recipients treated with IL-2cplx protocol are indicated in red, untreated are shown in grey. **(C)** Frequency of CD4+ (left) and CD8+ T cells (right) infiltrating the graft d6 post transplantation. **(D)** Frequency of CD4+ (left) and CD8+ (right) effector memory T cells (CD44+ CD62L-) found in skin allografts day 6 post transplantation. **(E)** Proportion of regulatory T cells (CD45+ CD4+ Foxp3+ CD25+) in the CD45+ CD4+ T cell population found within transplanted skin allografts day 6 after transplantation (left). Representative contour plot for regulatory T cell frequency within CD45+ CD4+ graft infiltrating cells (data from IR skin allograft transplanted mice; right). Analysis **(A–E)** was performed using flow cytometry and mean percentages of two independent experiments of untreated (IR: n = 5; non-IR: n = 5) and IL-2cplx protocol treated (IR: n = 5; non-IR: n = 6) recipients transplanted with irradiated or non-irradiated skin grafts are shown. Error bars indicate SD. (ns, not significant P > 0.05; *P < 0.05; **P < 0.01; two-tailed t test with unequal variances).

To study the immunological mechanisms leading to graft rejection, graft-infiltrating leucocytes (GILs) were investigated starting at day 6 after skin transplantation. Analysis of GILs in untreated recipients (wild type C57BL/6 grafted with BALB/c skin) revealed ~2.5-fold higher CD45+ cell frequencies in grafts previously exposed to IR in comparison to naïve (non-IR) grafts (mean 80% IR *vs* 32% non-IR; p = 0.02; **Figures 2A, B**). Based on staining for donor H-2Dd, nearly all (~98%) of the cells in the grafts at day 6 were of recipient origin (**Supplementary Figure 1**). Hence, prior IR of the donor grafts potentiated an early influx of host-derived inflammatory cells into the grafts (19). The GILs at day 6 included host CD4+ and CD8+ T cells (**Figure 2C**) which were enriched for cells with an effector phenotype (CD44+ CD62-) (**Figure 2D**). Within the CD4+ and CD8+ T cell compartment, however, IR grafts show an increase in naïve T cells, although it was significant only in CD4+ cells (**Supplementary Figure 2A**). However, IR did not lead to an influx of Tregs (**Figure 2E**). Notably, the results were substantially different when the hosts were treated with our tolerogeneic IL-2cplx protocol (**Figure 1A**) (20). Here, IL-2cplx treatment considerably reduced the influx of recipient leucocytes into the grafts and led to comparable low levels of GILs for both IR and normal (non-IR) grafts. This finding applied to CD45+ cells and conventional T cell subsets but, notably, not to Tregs. In fact, for Tregs there was a marked increase in migration of host Tregs into the grafts, both for normal and IR grafts (mean 38% IL-2cplx non-IR *vs*. 42% IL-2cplx IR; **Figure 2E**). This finding confirmed our previous observation that IL-2cplx-expanded Tregs can traffic rapidly into skin allografts (20). In addition, however, the data indicated that the influx of Tregs prevented the selective influx of other leukocytes into the IR grafts, consistent with the well-documented anti-inflammatory function of Tregs.

### Passenger Leucocyte Depletion Leads to Reduced Infiltration of Effector T Cells

**Figure 3:**
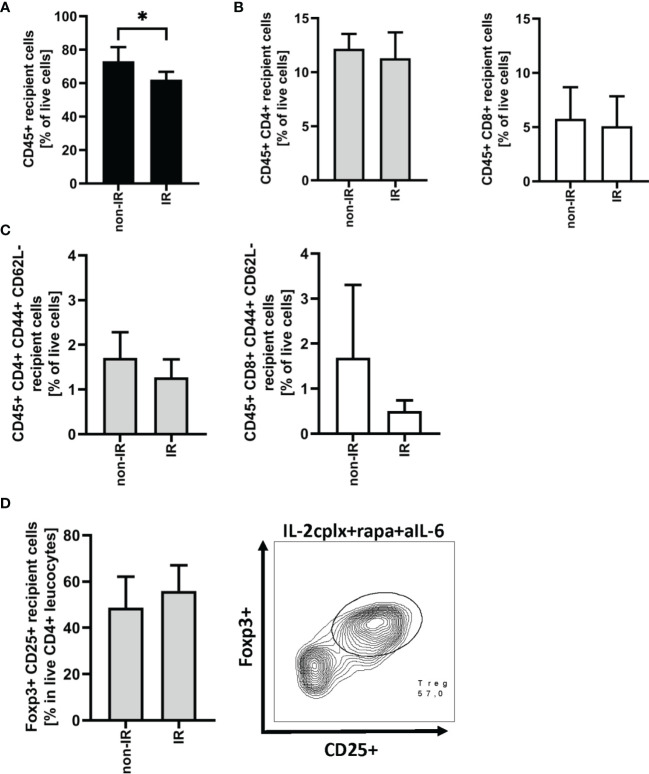
Graft infiltrating leucocytes (recipient) 20 days after transplantation. **(A)** Frequency of CD45+ graft infiltrating leucocytes within BALB/c skin grafts that were irradiated (IR) or non-irradiated (non-IR) before transplantation onto IL-2 complex protocol treated C57BL/6 recipients (IR: n = 6; non-IR: n = 7). **(B)** Frequency of CD4+ (left) and CD8+ (right) allograft infiltrating leucocytes. **(C)** Proportion of CD4+ (left) and CD8+ (right) effector memory T cells found within previously irradiated or non-irradiated skin allografts 20 days after transplantation. **(D)** Percentage of regulatory T cells (CD45+ CD4+ Foxp3+ CD25+) in the CD45+ CD4+ T cell population present within the skin graft 20 days after transplantation (left). Representative contour plot of regulatory T cell frequency within CD45+ CD4+ graft infiltrating cells if recipient was treated with IL-2cplx protocol (right). Analysis **(A–D)** was performed using flow cytometry and mean percentages of two independent experiments of IL-2cplx protocol treated recipients transplanted with irradiated (n = 6) or non-irradiated (n = 7) skin graft are shown. Error bars indicate SD. (*P < 0.05; two-tailed t test with unequal variances).

As discussed below, prolonged survival of skin allografts was seen only in IL-2cplx-treated mice. Hence, examination of GILs at late stages of engraftment was restricted to the two groups of IL-2cplx-treated mice. Data on cells recovered from the grafts on day 20 are shown in **Figure 3**. At this time point, no macroscopic differences of skin grafts were visible. However, the percentage of CD45+ leukocytes was significantly (~20%) higher in normal than in IR grafts (mean 62% IR *vs* 73% non-IR; p = 0.01; **Figure 3A**). For CD4+ and CD8+ cells, there was also a shift towards a higher percentage of T effector cells in the IR grafts (**Figures 3B, C**; **Supplementary Figure 2B**). Comparably high Treg frequencies (~50% of CD4+ T cells) were found within IR and non-IR skin grafts, suggesting active regulation in both groups (**Figure 3D**).

### Reduction of Passenger Leucocytes Leads to Prolonged Skin Allograft Survival in IL-2cplx Protocol Treated Mice

**Figure 4:**
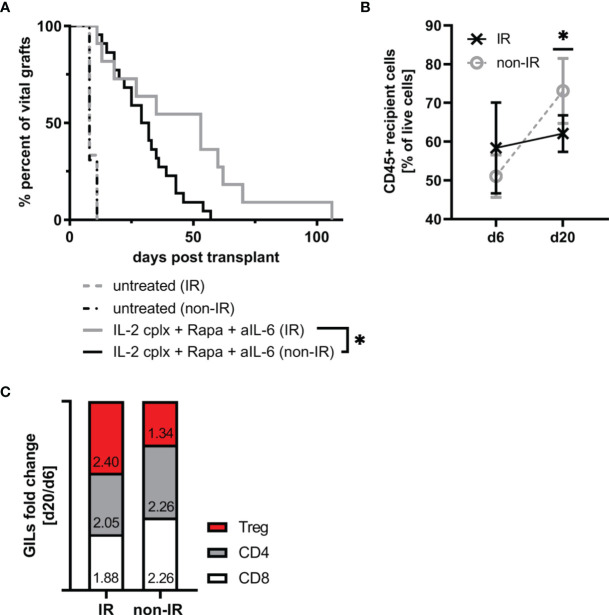
Irradiation of donor skin graft prior to transplantation leads to significant prolongation of skin allograft survival in combination with IL-2cplx protocol treatment (MST: IR=53 days; non-IR=30.5 days; p = 0.0192, log-rank test). **(A)** Survival of irradiated (IR) and non-irradiated (non-IR) skin grafts on mice treated with (IR: n = 8; non-IR: n = 8) or without (IR: n = 11; non-IR: n = 22) a combination of IL-2 complexes, Rapamycin and a short term treatment of anti-IL-6. Survival proportions of at least two independent experiments are shown. **(B)** Mean frequency of CD45+ graft infiltrating leucocytes on day 6 and day 20 after transplantation in irradiated (d6: n = 3; d20: n = 6) and non-irradiated (d6: n = 6; d20: n = 7) skin allografts transplanted on IL-2cplx protocol treated recipient mice. **(C)** Differences in fold change between d6 and d20 post transplantation in non-irradiated (left; d6: n = 6; d20: n = 7) and irradiated (right; d6: n = 3; d20: n = 6) skin allografts on IL-2cplx protocol treated recipient mice. Calculation: Fold change was obtained by dividing the mean value of two independent experiments for d20 by the mean value of d6. Fold changes were normalized to values obtained for non-irradiated skin grafts (shown as baseline with value 0). Analysis **(B, C)** was performed using flow cytometry and mean percentages of two independent experiments are shown. (*P < 0.05; two-tailed t test with unequal variances).

Since the IR grafts were largely depleted of APC at the time of grafting, we expected survival of these grafts to be significantly prolonged. For the groups that were not IL-2cplx treated, however, this was not the case. Thus, both the control non-IR and IR grafts were rejected rapidly at the same time, 8 days after transplantation (**Figure 4A**). With the IL-2cplx-treated groups, the results were different. Here, as expected, IL-2cplx treatment led to prolonged survival of both the control and IR grafts. Notably, however, the IR grafts survived for around twice as long as the control grafts (MST IR = 53 days; non-IR = 30 days; p = 0.02), (**Figure 4A**).

These data indicate therefore that the capacity of IL-2cplx treatment to enhance allograft survival could be further improved by prior irradiation of the grafts. With regard to the mechanisms involved, for the control non-IR grafts the frequency of CD45+ cells infiltrating the grafts increased significantly between day 6 and day 20 day after transplantation (mean 51% d6 *vs* 73% d20; p = 0.001). For the IR grafts, by contrast, levels of CD45+ cells did not increase between day 6 and day 20 (mean 58% d6 *vs* 62% d20) (**Figure 4B**). Nevertheless, during this time there was a subtle change in the proportions of T cell subsets in the grafts. Thus, when fold changes of graft-infiltrating T cell subsets between days 6 and 20 were compared, the IR grafts showed a selective enrichment of Tregs relative to normal CD4 and CD8 T cells (**Figure 4C**). Hence, based on the GILs examined at these time points, the enhanced survival of the IR grafts correlated with a shift from conventional T cell subsets to Tregs in the grafts.

### Reduction of Passenger Leucocytes Does Not Affect Donor-Specific Humoral Response

**Figure 5:**
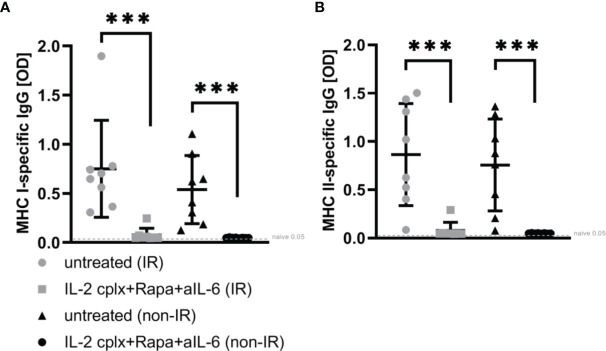
ELISA for donor-specific antibodies within blood sera 14 days post skin graft rejection. Irradiated (IR) or non-irradiated (non-IR) skin allograft recipients (C57BL/6) were either left untreated or IL-2 complexes in combination with rapamycin and anti-IL-6 (short term) were administered. Blood sera isolation was performed d14 post rejection of BALB/c skin allograft and assessment of donor specific IgG (IgG1, IgG 2a/b, IgG3) against **(A)** MHC class I (H2k-d) and **(B)** MHC class II (IA-d) was done utilizing ELISA. Mean percentages of at least two independent experiments are shown. Error bars indicate SD. (***p < 0.001; two-tailed t test with unequal variances) (untreated (IR): n = 8; untreated (non-IR): n = 8; IL-2cplx protocol (IR): n = 8; IL-2cplx protocol (non-IR): n = 8).

To determine whether the absence of passenger leucocytes also affects humoral responses, serum of mice grafted with IR or non-IR skin was investigated day 14 post rejection of skin allografts. Donor H2k-d (MHC I) and IA-d (MHC II) specific ELISA showed that recipient mice receiving IL-2cplx do not develop donor specific IgG responses (**Figures 5A, B**). This was seen for MHC I as well as MHC II and was independent of whether IR or non-IR skin allografts were applied, suggesting that the absence of passenger leucocytes has no direct influence on the development of anti-donor antibodies.

## Material And Methods

### Mice

Female C57BL/6 (recipient; H-2^b^), BALB/c (donor; H-2^d^) and C3H (H-2^k^) mice were purchased from Charles River Laboratories (Sulzfeld, Germany) and housed in ventilated HEPA-filtered cages at the Medical University of Vienna under specific pathogen-free like conditions. Mice were used for experiments between 6 and 8 weeks of age with an average weight of 18 to 21 g. All experiments were approved by the ethics votum of the Austrian Federal Ministry of Science, Research and Economy (Permission number GZ: BMWFW-66.009/0292-V/3b/2018).

### Preparation of IL-2/anti-IL-2 mab Complexes (IL-2cplx)

IL-2 complexes were prepared as previously described (20, 21). Briefly, IL-2/anti-IL-2 complexes were prepared by mixing recombinant mouse IL-2 (PeproTech) with anti-IL-2 mab (clone JES6-1A12, BioXCell) in a 1:5 ratio and incubating at 37°C for 30 min. Mice received a final volume of 300 μl intraperitoneally (i.p.).

### Generation of Skin Resident Leucocyte Reduced Donor Mice

Donor mice (BALB/c) were subjected to lethal total body irradiation (TBI) of 8.5 Gy at day -8 and were reconstituted with 10 x 10^6^ bone marrow cells of naïve BALB/c mice at day -7. Skin graft donation followed 8 days post TBI.

### IL-2 Complex Protocol

The IL-2 complex protocol was administered as previously described (20). Briefly, indicated groups of age-matched C57BL/6 mice received IL-2 complexes (1 μg IL-2/5 μg JES6-1A12 i.p.) on 3 consecutive days starting on day 3 before transplantation, which was continued to day 1 after transplantation with injections thrice a week until day 30. In addition, rapamycin (1 mg/kg i.p., LC Laboratories) was administered day -1/0/1 followed by injections thrice a week until day 30. Furthermore, mice received a short-term treatment with anti-IL-6 mab (clone MP5-20F3, BioXCell) at days -1/1/4/6 (300 μg i.v.).

### Skin Grafting

Full thickness tail skin of naïve BALB/c (donor) mice or BALB/c mice previously subjected to lethal TBI and reconstituted with isogenic BM were grafted on the flank (lateral thorax wall) of naïve C57BL/6 (recipient) mice. After transplantation skin grafts were secured with band aids for 6 days, followed by visual inspections at short intervals. Skin grafts were considered rejected if less than 10% remained viable. During transplantation mice were anesthetized with Ketanest (Ketamin, 100 mg/kg) and Rompun (Xylazine, 5 mg/kg). Postoperatively mice received Temgesic (Buprenophin, day 0; 0,01 – 0,05 mg/kg i.p.) and Dipidolor (Piritramide, 15 mg in 250 ml 0,4% glucose water) in drinking water ad libitum for 1 week.

### Flow Cytometric Analysis and Antibodies

For characterization of leucocyte subtypes antibodies against mouse CD45.2 (104), H2D^d^ (34-2-12), CD3 (17A2), CD4 (RM4-5 and GK1.5), CD8 (53-6.7), CD44 (IM7), CD62L (MEL-14), CD25 (PC61.5), CD80 (16-10A1), CD86 (PO3), CD11b (M1/70), CD11c (N418), MHC II (M5/114.15.2), 7AAD (viability staining solution, purchased from BioLegend), Foxp3 (FJK-16s) and fixable viability dye (Fixable Viability Dye eFlour 450 and 506 purchased from eBioscience) were used. Cell suspensions were stained for surface markers for 30 min., 4°C in the dark. Intracellular Foxp3 staining was done using a Fixation/Permeabilization kit (eBioscience) according to the manufacturer’s instructions. Erythrocyte lysis in spleen cells was performed utilizing red blood cell lysis buffer (Sigma). Analysis was done with BD FACS Canto II and FlowJo software.

### Isolation of Graft-Resident/Graft Infiltrating Leucocytes (GILs)

Skin grafts were cut into small pieces in RPMI medium before digestion using a murine Tumor Dissociation Kit (Miltenyi Biotec) according to the manufacturer`s instructions. Cells were resuspended in cold PBS and the number of viable cells was determined using a CASY cell counter (Innovatis) before flow cytometrical staining. GILs were defined as viable single cells expressing CD45.

### Enzyme-linked Immunosorbent Assay (ELISA)

To detect IgG and IgM against donor MHC I (H2k-d) and MHC II (IA-d), a donor MHC-specific ELISA was performed as previously described (22). Briefly, a 96-well plate was coated with 5µg/mL of H2K^d^ or I-A^d^ monomers at 4°C, overnight. Serum samples obtained 14 days post rejection of skin allografts were diluted 1:100 before incubation with monomers at 4°C, overnight. Donor-specific antibodies bound to monomers were detected utilizing monoclonal rat anti-mouse IgG1, IgG2a/b, IgG3 in combination with an HRP-coupled goat anti-rat serum (1:2000). ABTS was used as substrate for HRP and absorption was measured using a Victor microplate reader (405nm). The biotinylated MHC monomers were kindly provided by the National Institutes of Health’s Tetramer Core Facility (https://www.niaid.nih.gov/research/nih-tetramer-core-facility).

### Statistics

The statistical analysis was performed using GraphPad Prism 5.0 (GraphPad Software, La Jolla, CA, USA). Error bars indicate standard deviation (SD) and differences between groups were compared using a 2-tailed Student´s t-test with unequal variances. Survival of skin allografts was calculated based on Kaplan-Meier product limit method and compared between groups using the log-rank test. Fold change in GILs was calculated dividing the mean value of two independent experiments for d20 by the mean value of d6. A *p*-value < 0.05 was considered statistically significant.

## Discussion

The role of passenger leucocytes and its impact on graft survival after solid organ transplantation is complex. Although several groups demonstrated increased survival of mismatched organs depleted of graft-resident cells in rodent models (5-8), there is also contrary evidence suggesting beneficial immunomodulatory effects of passenger leucocytes in transplantation (17). Studies on the long-term tolerance of allogeneic liver in rats, for example, revealed an essential function of donor-derived intrahepatic leucocytes on graft survival (23). Furthermore, using a rodent model for fully MHC-mismatched heart transplantation, tolerance induction was prevented if donor leucocytes were depleted on the day of transplantation using a monoclonal antibody against donor-specific CD45-expressing cells (24). In the study presented herein, we used a tolerogeneic treatment regimen based on IL-2cplx injection to demonstrate that the survival of fully MHC-mismatched skin grafts is significantly prolonged if donor-derived leucocytes are reduced. These data thus support the hypothesis that passenger leucocytes promote the rejection of allogeneic skin grafts.

Contact with DCs is crucial for immune responses after solid organ transplantation with DCs being 100 times more potent in activating T cells than MHC II-bearing macrophages or B cells (25). Within the skin, two types of resident DCs are well characterized: Dermal DCs and epidermal Langerhans cells (LC) (26). Besides being professional and efficient APCs, LCs of the epidermis are suggested to be effective in stimulating effector responses resulting in graft rejection, i.e. inducing naïve CD4+ T cell differentiation into effector phenotypes (27–29) or priming of naïve CD8+ T cells (30). We could show that irradiation of donor mice (8 days prior to tail skin donation) leads to substantial reduction of these graft-resident APCs, in accordance to what has been previously shown for dermal DCs and epidermal LCs in murine ear skin (18). In addition, we were able to demonstrate IR-related effective reduction of CD4+ as well as CD8+ T cells within the donor tail skin. The reduction and depletion of donor leucocytes not only decreases donor antigen-dependent recipient (direct) T cell allo-recognition (31), but also reduces donor-derived CD4+ T cell-related increase of recipient immune responses leading to early graft failure (32). Besides, using this approach we were able to deplete graft-resident leucocytes without altering the host´s immune system, creating an eligible model for analysis of allo-recognition mechanisms.

At face value, the finding that the IR grafts were rapidly rejected on normal hosts would seem to argue against the concept that removal of passenger leukocytes improves graft survival. However, as demonstrated here the IR grafts were rapidly infiltrated with host T cells. Thus, at day 6 after transplantation the frequencies of recipient CD4+ and CD8+ effector T cells were exceptionally high in skin grafts previously subjected to IR, suggestive of active inflammation. This finding is in line with literature showing that IR leads to the release of pro-inflammatory cytokines and chemokines as well as oxidative stress (19). Hence, the enhanced influx of host T cells into the IR grafts would be expected to augment the rejection process. Our suggestion therefore is the failure of passenger leukocyte depletion to delay rejection of the IR grafts was largely due to irradiation-induced inflammation causing an enhanced host-*versus*-graft response. This notion is in line with the report that early infiltration of donor-reactive CD8+ T cells promoted fast allograft rejection *via* IFNγ production in a cardiac transplant model (33). However, we cannot exclude the possibility that the fully-mismatched IR grafts were less immunogenic in terms of long-term sensitization. Relative to non-IR grafts, the IR grafts elicited lower levels of memory-phenotype T cells after rejection, though whether these cells were antigen-specific was unclear (34). Studies on the kinetics of second-set rejection are in progress. Importantly, for both grafted groups Treg numbers soon returned to the normal levels seen in naïve C57BL/6 mice, confirming that there are no unspecific long-term immunosuppressive effects due to IL-2cplx treatment (unpublished data). Overall, the data provide further support for the view that passenger leukocytes play a significant though not obligatory role in allograft rejection. Definitive information on which particular subsets of skin APC are needed for sensitization will require studies on the selective depletion of DC, LC and related cells (35).

Here, we could show that in mice receiving tolerogeneic IL-2cplx treatment, the reduction of graft-resident leucocytes leads to significant increase of skin allograft survival, illustrating the effect of graft-resident cells on long-term transplant acceptance. As already mentioned, irradiation induces tissue damage and inflammation independent from allo-specific responses. Treg expansion induced by IL-2cplx treatment leads to an anti-inflammatory, suppressive environment, both within the graft and in the host lymphoid tissues. We suggest that under tolerogeneic conditions the decreased immunogenicity of leucocyte depleted grafts favors improved long-term survival.

We also analyzed donor-specific antibody (DSA) formation since DSAs are known to be one major issue in graft injury and chronic rejection (36). As previously demonstrated by our group DSA IgG development is impaired if recipients are treated with IL-2cplx protocol (20). In line with this, we could show that IgG-related humoral immunity was almost absent in the recipients depleted of graft-resident leucocytes suggesting that passenger leucocytes seem to be important in T cell priming but do not influence GC formation or antibody production.

Although this study is restricted to skin allograft transplantation, it clearly demonstrates that the absence of graft-resident cells (in this study achieved by IR pre-treatment of the donor) is beneficial for long-term transplant survival if early IR-related pro-inflammatory processes are overcome. As already mentioned IR is accompanied by a variety of side effects (19), discouraging clinical translation. Alternative approaches for reduction/depletion of passenger leucocytes are based on graft-specific modifications using i.e. RNA interference for prevention of donor cell trafficking (CCL7) or suppression of donor-derived EV release may have superior potential for future translation to the clinical setting (12, 37). Since the discovery of RNA interference (38) promising data have been obtained in preclinical studies of rodent heart and kidney models utilizing small interference RNA (siRNA) targeting complement system or blocking co-stimulation with successful prolongation of graft survival (39–41). More importantly, there are ongoing phase 3 clinical trials involving siRNA in kidney transplantation with evidence of successful prevention of acute kidney injury and delayed graft function (42–44). By use of perfusion machines, donor leucocytes are mobilized into the perfusate, which allows their removal with a leucocyte filter before transplantation (45). Normothermic machine perfusion including a leucocyte filter was shown to reduce acute rejection and T cell priming in a porcine lung transplantation model (46). Future approaches could include *ex vivo* organ perfusion with depleting antibodies or anti-thymocyte globulin (ATG) to reduce the number of specific graft-resident leucocyte subsets within the graft.

In this proof-of-principle study we could demonstrate the importance of donor-derived leucocytes in recipient sensitization and allograft rejection, which may lay the ground to new translational approaches. Graft survival and long-term outcome could be improved by targeting early allogeneic responses and acute rejection episodes due to rapid recognition of allogeneic MHC in direct T cell allorecognition. Studies investigating the effect on long-term survival and chronic rejection in primarily vascularized grafts are clearly warranted.

More importantly, these data suggest that treatment with the IL-2cplx protocol ameliorates (non-allogeneic) inflammatory processes caused by e.g. IR and therefore counteracts the early augmented infiltration of leucocytes into the graft, which could trigger acute rejection and early graft loss.

## Data Availability Statement

The original contributions presented in the study are included in the article/**Supplementary Material**. Further inquiries can be directed to the corresponding author.

## Ethics Statement

The animal study was reviewed and approved by Federal Ministry Republic of Austria Education, Science and Research.

## Author Contributions

All authors have actively contributed to this project. RS and NP designed all experiments and analyzed data. RS, NP, and AW performed experiments. RS, NP, JS, and TW interpreted data and wrote the manuscript. All authors contributed to the article and approved the submitted version.

## Funding

This work was supported by the Austrian Science Fund (J3671-B19 and P31186-B28 to NP) and grants from the National Health and Medical Research Council of Australia to JS.

## Conflict of Interest

The authors declare that the research was conducted in the absence of any commercial or financial relationships that could be construed as a potential conflict of interest.

## Publisher’s Note

All claims expressed in this article are solely those of the authors and do not necessarily represent those of their affiliated organizations, or those of the publisher, the editors and the reviewers. Any product that may be evaluated in this article, or claim that may be made by its manufacturer, is not guaranteed or endorsed by the publisher.
