# Supplementary material for: Impact of Graft-Resident Leucocytes on Treg Mediated Skin Graft Survival
Source: Front Immunol. 2021 Nov 29;12:801595. doi: 10.3389/fimmu.2021.801595 (PMC8666425; doi:10.3389/fimmu.2021.801595)
Supplement: Supplementary file 1 [file DataSheet_1.docx]

**Supplementary Figure 1: Donor-specific leucocytes within skin allografts day 6 after transplantation.** **(A)** Frequencies of viable donor leucocytes (34-2-12 positive CD45 positive) remaining within skin grafts 6 days after transplantation. In addition, proportion of donor-derived CD4+ as well as CD8+ T cells within graft resident leucocytes is shown. Analysis was performed using flow cytometry and mean percentages of two independent experiments are shown. **(B)** Representative histogram for viable skin allograft resident donor-specific leucocytes (34-2-12 positive CD45 positive; top figure). Furthermore, representative histograms for donor-specific (34-2-12 positive) CD4+ (left) and CD8+ (right) T cells within skin allografts day 6 after transplantation.

**Supplementary Figure 2:**  **Recipient T cell subsets infiltrating irradiated (IR) and non-irradiated (non-IR) skin allografts over time.** **(A)** Proportion of graft infiltrating naïve (CD44- CD62L+) and effector (CD44+ CD62L-; Teff) recipient T cells day 6 after transplantation. Host mice were treated with a combination of IL-2 complexes (IL-2cplx) , rapamycin and short-term anti-IL-6 (IR: n=5, non-IR: n=6) or left untreated (IR: n=5, non-IR: n=5). **(B)** Frequency of recipient naïve (CD44- CD62L+) and effector (CD44+ CD62L-; Teff) T cells infiltrating a IR (n=6) or non-IR (n=7) skin graft transplanted on host mice treated according to IL-2cplx protocol (untreated mice already rejected graft at this time point). Analysis (A, B) was performed using flow cytometry and mean percentages of two independent experiments are shown. Error bars indicate SD. (NS, not significant P > 0.05; *P < 0.05; two-tailed *t* test with unequal variances)
