# Supplementary figures and images for: Impact of Graft-Resident Leucocytes on Treg Mediated Skin Graft Survival
Source: Front Immunol. 2021 Nov 29;12:801595. doi: 10.3389/fimmu.2021.801595 (PMC8666425; doi:10.3389/fimmu.2021.801595)

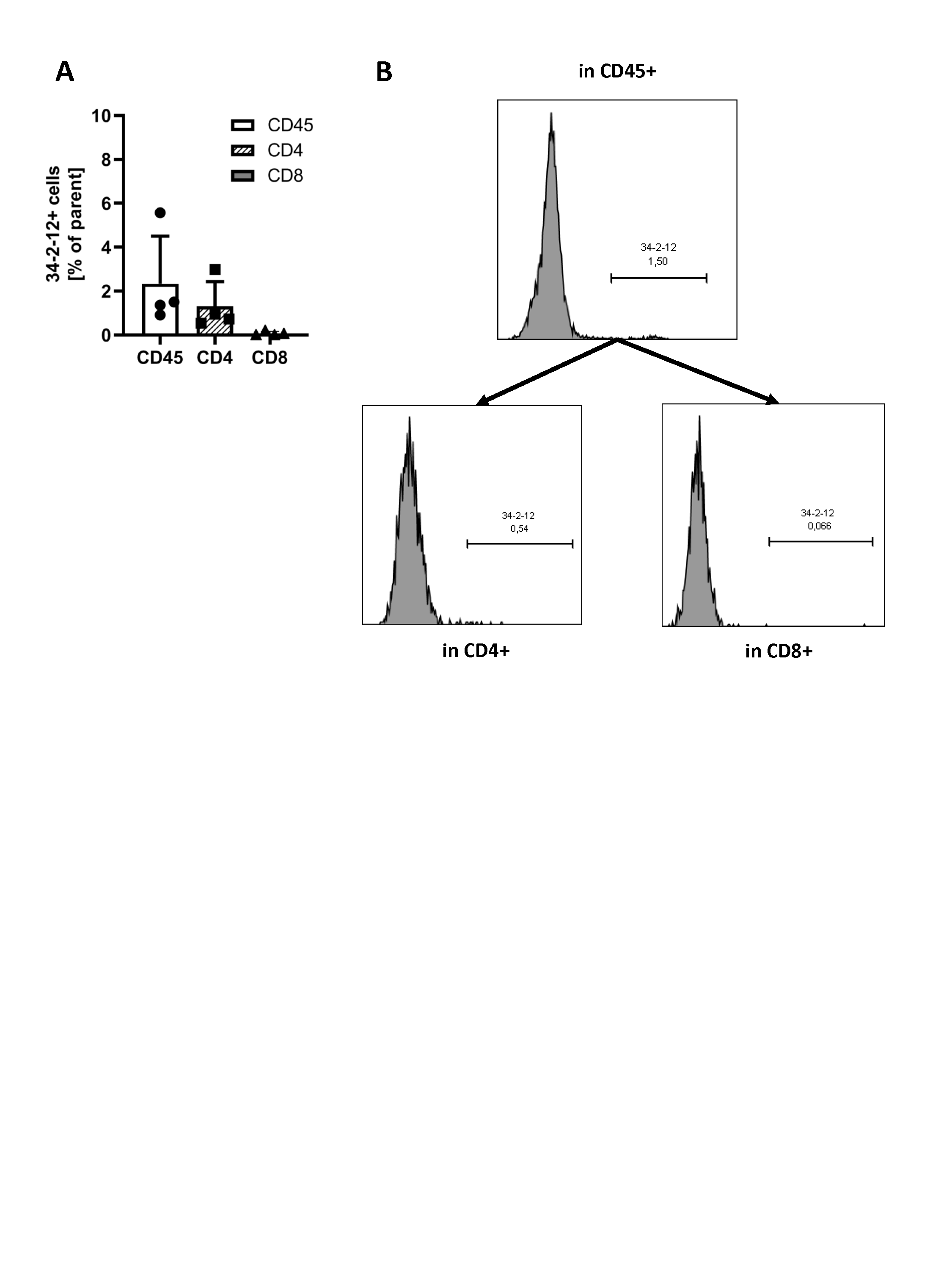

Supplement: Supplementary file 2 [file Image_1.tiff]

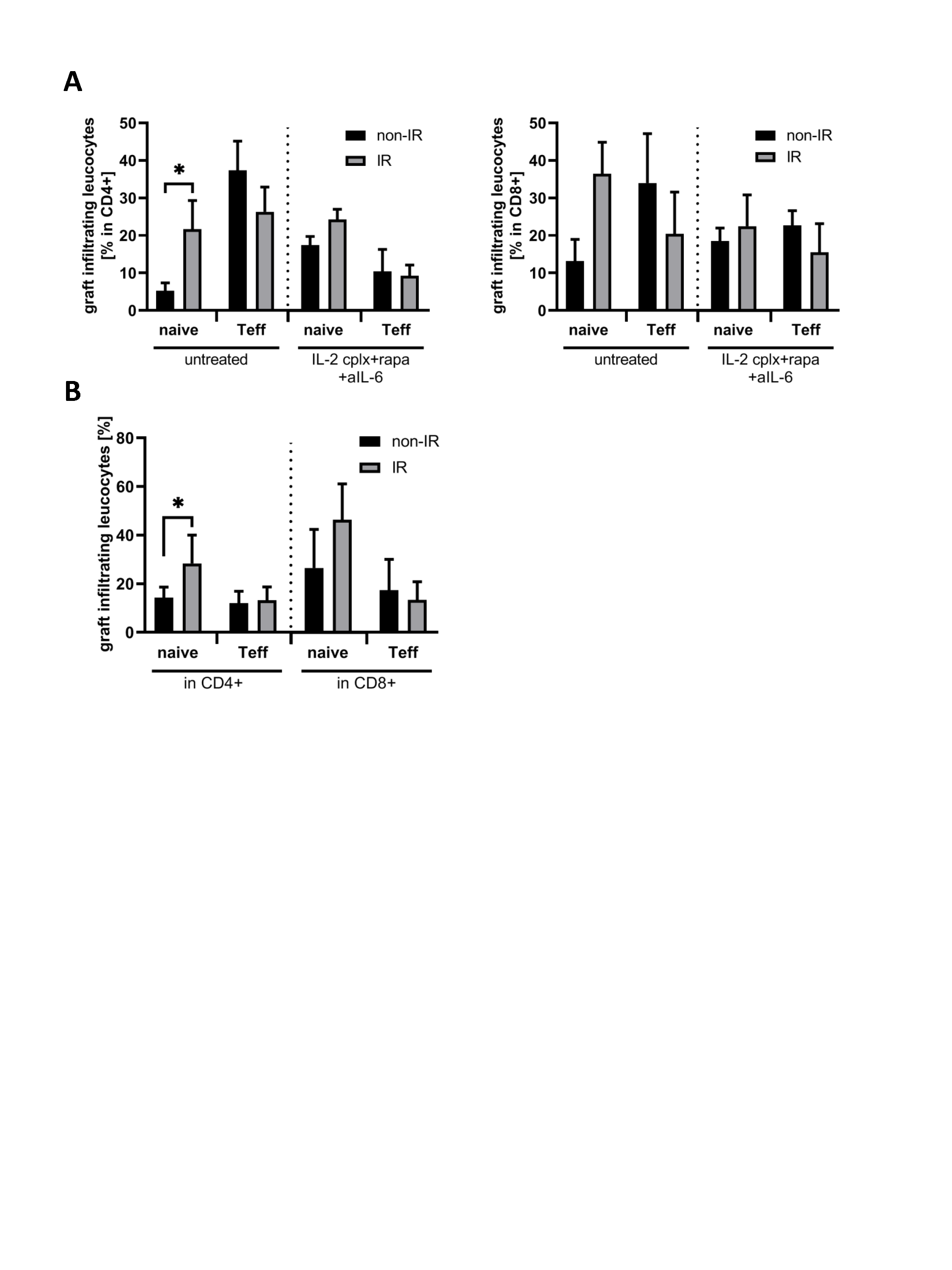

Supplement: Supplementary file 3 [file Image_2.tiff]
